# Advancing reproductive health on the humanitarian agenda: the 2012-2014 global review

**DOI:** 10.1186/1752-1505-9-S1-I1

**Published:** 2015-03-26

**Authors:** Sarah K Chynoweth

**Affiliations:** University of New South Wales, High St, Kensington, NSW 2052 Australia

## Introduction

**Figure 1:**
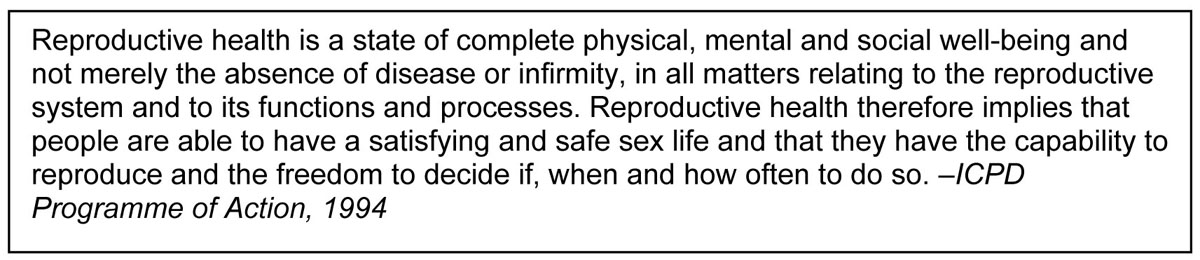
Figure 1

The global landscape for reproductive health in humanitarian settings has changed dramatically since the International Conference on Population and Development (ICPD) in 1994. Mainstreaming of reproductive health into humanitarian health responses has grown, and awareness of the consequences of neglecting reproductive health services, such as maternal and neonatal mortality, HIV transmission, and unsafe abortion, has expanded. Despite these advances, significant gaps remain, and meeting the reproductive health needs of crisis-affected communities is more urgent than ever: the United Nations High Commissioner for Refugees (UNHCR) reported that 51.2 million people remained forcibly displaced due to conflict and persecution by the end of 2013—the largest number since World War II [1]. An additional 22 million were displaced in 2013 by natural disasters [2]. Figure 1.

A concentrated effort to address reproductive health in emergencies commenced in 1995 when a coalition of UN agencies, national and international nongovernmental organizations (NGOs), government agencies, donors, and academic institutions established the Inter-agency Working Group on Reproductive Health in Crises^a^ (IAWG), an international network dedicated to improving the reproductive health of communities affected by conflict and natural disaster. IAWG arose from a growing concern with the lack of attention to reproductive health, despite increasing evidence of its need in emergency settings [3]. In its first decade, IAWG made large strides in advancing reproductive health through advocacy, research, standard setting, and guidance development, including the publication of the seminal *Reproductive Health for Refugees: An Inter-agency Field Manual* [4]. The *Field Manual* was the first technical guidance on implementing reproductive health in emergencies and articulated a minimum standard in reproductive health service delivery—the Minimum Initial Service Package (MISP) for Reproductive Health.^b^ IAWG also supported the creation of the Inter-agency Reproductive Health Kits, twelve kits of essential medicines and supplies, to support rapid implementation of the MISP [5].

By the early 2000s, IAWG and its partners—including the Reproductive Health Response in Crises (RHRC) Consortium^c^—had achieved substantial gains. A 1999 study documented an increase in evidence, funding, policies, conferences, and new NGOs addressing reproductive health in emergencies, reflecting marked progress in advancing reproductive health on the global humanitarian agenda [6]. From 2002 to 2004, IAWG undertook its first global evaluation to assess progress [7]. The findings confirmed advancements at the policy and implementation levels since the mid-90s, but significant gaps continued across all technical areas, specifically maternal and newborn health, family planning, gender-based violence, and HIV and other sexually transmitted infections (STIs).

IAWG’s second decade, from 2004 to 2014, saw the maturation of the coalition and further advancements to institutionalize reproductive health into humanitarian health responses and improve access to services. Members successfully advocated integrating the MISP as a minimum health standard in the 2004 and 2011 revisions of the *Sphere Humanitarian Charter and Minimum Standards in Disaster Response* and the *Inter-Agency Standing Committee Health Cluster Guide*[8, 9]. Through IAWG’s advocacy, the MISP was included as a life-saving activity eligible for Central Emergency Response Fund funding [10]. In 2009, led by the World Health Organization (WHO) and UN Population Fund (UNFPA), IAWG and partners drafted the *Granada Consensus on Sexual and Reproductive Health during Protracted Crises and Recovery*, which reaffirmed comprehensive reproductive health as a right in protracted settings and fragile states [11]. The following year IAWG released an updated field-test version of the *Field Manual*, which included an extra chapter dedicated to comprehensive abortion care—a particularly neglected area in reproductive health service provision—as well as outlined additional priority activities to the MISP [12]. IAWG also served as a platform to spearhead two ground-breaking, complementary programs: the Reproductive health Access, Information and Services in Emergencies (RAISE) Initiative, which focuses on expanding comprehensive reproductive health services in crises, and the Sexual and Reproductive Health Programme in Crisis and Post-Crisis Situations (SPRINT), which works to enhance access to the priority services of the MISP. These initiatives are among the first international efforts to systematically scale up capacity and implementation of reproductive health services in emergencies at the national level.

Membership expanded as IAWG actively sought to decentralize and establish regional networks. By the end of 2014, IAWG had 1,680 individual members representing 124 countries and 450 different agencies—a significant increase from approximately 50 members in 2004. With more members, IAWG was able to establish regional chapters as well as roughly ten sub-working groups on specialized issues related to reproductive health, such as new technologies, urban displacement, and disaster risk reduction. Indeed, IAWG’s disaster risk reduction and emergency preparedness efforts, including the SPRINT Initiative and the reproductive health group within the UN International Strategy for Disaster Reduction, have helped promote a comprehensive approach to reproductive health that considers both pre- and post-crisis phases. IAWG has galvanized the field despite lack of sustained, dedicated funding since the coalition’s inception.

**Figure 2:**
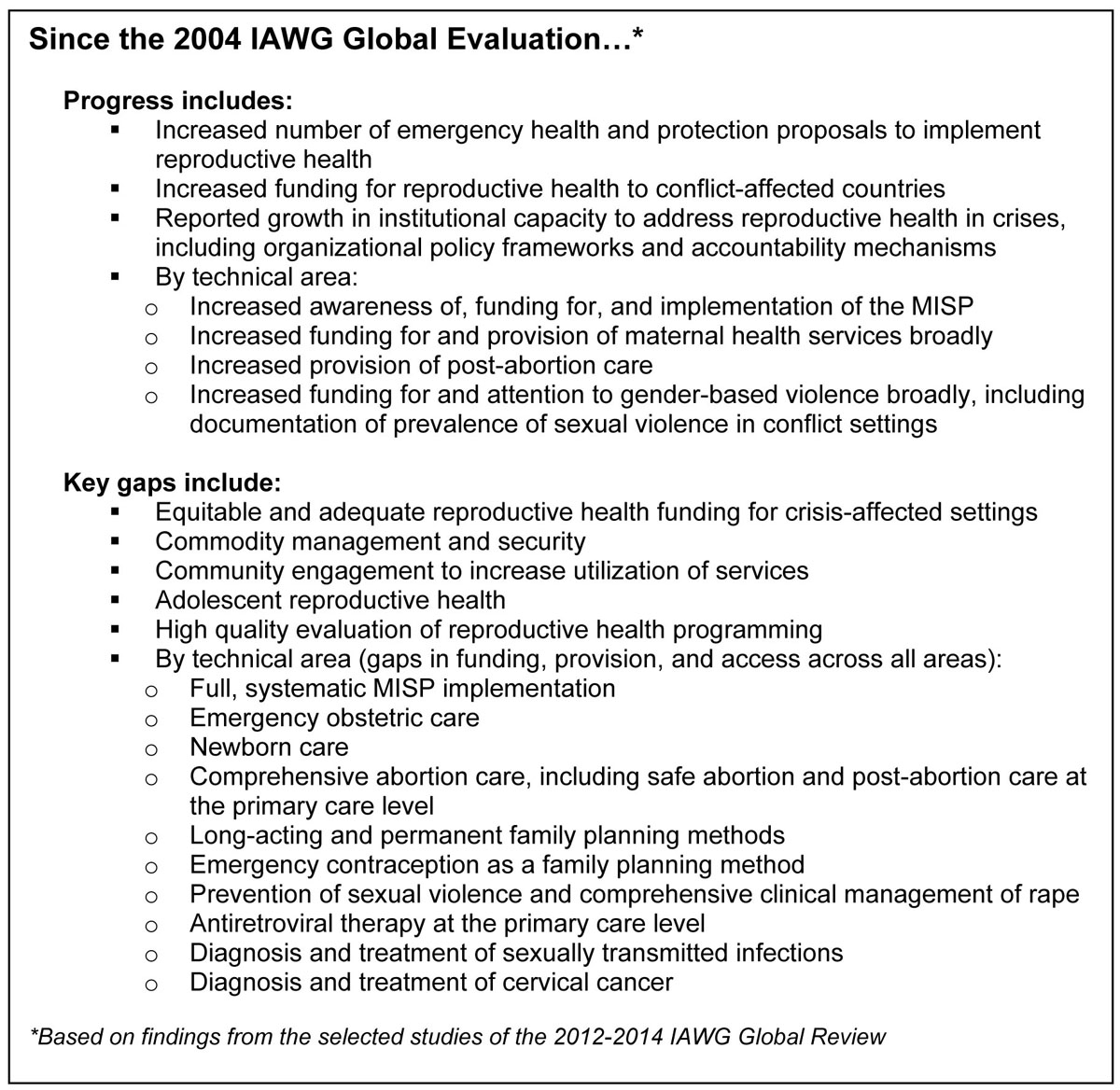
Figure 2

From 2012 to 2014, IAWG conducted a second global review to assess progress, document gaps, and determine future directions. Seven complementary studies were undertaken to provide a snapshot of the field. The studies build on those undertaken for the 2004 evaluation and explore key aspects of the field, including new research, changes in funding and institutional capacity, and implementation of both MISP and comprehensive reproductive health services in selected settings. Four studies are presented in this *Supplement*: a systematic review of peer-reviewed research evaluating reproductive health programs in crises from 2004 to 2013 [13]; an assessment of MISP implementation in two settings hosting Syrian refugees in Jordan [14]; an evaluation of the availability and quality of and access barriers to reproductive health services in crisis-affected settings in Burkina Faso, the Democratic Republic of the Congo (DRC), and South Sudan [15]; and a systematic analysis of reproductive health in humanitarian health and protection funding proposals for 2002 to 2013 [16]. Three additional studies, not yet published, include a long-term trend analysis study that tracked official development assistance for reproductive health to 18 conflict-affected countries for 2002 to 2011 (unpublished observations, Patel, Dahab, Tanabe, Murphy, Ettema, Guy, Roberts), a retrospective analysis of selected reproductive health indicators from UNHCR’s Health Information System across 56 refugee camps in ten countries from 2007 to 2013 (unpublished observations, Whitmill, Tomczyk, Blanton, Doraiswamy, Haskew, Cornier, Schilperood, Spiegel), and a survey of humanitarian and development agencies that explored changes in their capacity to address reproductive health in crises since 2004 (unpublished observations, Tran, Dawson, Meyers, Krause, Hickling). The findings revealed substantial progress since 2004—reproductive health is squarely situated on the humanitarian agenda—but multiple gaps were documented across all technical areas punctuated by overarching issues in commodity security and community engagement. Figure 2.

## Progress and gaps

Overall, the studies documented broad progress for reproductive health in humanitarian settings. Tanabe et al. found that of the roughly 11,300 health and protection proposals issued between 2002 and 2013, almost 4,000 contained reproductive health components, more than a third of the issued proposals [16]. The number of proposals including reproductive health increased by an average of 22% per year, while the proportion of health and protection sector proposals containing reproductive health increased by an average of 10% per year. Preliminary findings from the long-term trend analysis indicate substantial increase in official development assistance for reproductive health for conflict-affected countries from 2002-2011 (unpublished observations, Patel, Dahab, Tanabe, Murphy, Ettema, Guy, Roberts). These studies demonstrate increased awareness among humanitarian actors of the need to implement reproductive health services in a crisis response. The trend is also reflected by the preliminary findings of the institutional capacity survey that documented increased organizational investment in human and financial resources to address reproductive health in humanitarian settings (unpublished observations, Tran, Dawson, Meyers, Krause, Hickling).

At the same time, although requests for funding and absolute funding received for reproductive health in humanitarian appeals have increased since 2002, Tanabe et al. determined that just 43% of these funding requests were met over the last 12 years, slightly above the health sector average of 41% and well below the total humanitarian sector average of 68% [16]. Preliminary findings from the long-term trend analysis indicate that the bulk of increased overseas development assistance for reproductive health in conflict-affected countries was attributable to HIV-related activities (unpublished observations, Patel, Dahab, Tanabe, Murphy, Ettema, Guy, Roberts). The preliminary findings also show disparity in the disbursement of overseas development assistance for reproductive health between conflict-affected countries and non-conflict affected countries in the same income category and between countries affected by conflict; for example, the average annual per capita reproductive health overseas development assistance disbursed to least-developed non-conflict-affected countries was 57% higher than to least-developed conflict-affected countries.

### MISP

The MISP comprises the priority reproductive health activities to be implemented in an acute emergency and should be built upon with comprehensive programming as soon as possible. In 2004, the first global evaluation found that attention to the MISP was gaining ground, but implementation was not systematic, particularly during the early days of a response, and awareness among health actors remained low [7]. Over the past decade, assessments spearheaded by the Women’s Refugee Commission, UNFPA, and other IAWG members have chronicled steady improvement in MISP awareness and implementation globally [17–21]. For the 2014 global review, Krause et al. found services and key elements to support MISP implementation largely in place in two settings serving Syrian refugees in Jordan [14]; preliminary findings from the institutional capacity study show increasing attention to reproductive health coordination by the agencies surveyed (unpublished observations, Tran, Dawson, Meyers, Krause, Hickling). Tanabe et al. determined that, among humanitarian health and protection appeals submitted between 2009 and 2013, proposals that included all of the components of the MISP and those with partial MISP components increased an average of almost 40% and 2.4%, respectively, per year [16].

The other studies paint a more complex picture. Casey et al.’s research revealed significant gaps in the clinical components of the MISP in three settings well past the acute emergency phase [15]. Similarly, preliminary findings from the analysis of reproductive health indicators from UNHCR’s Health Information Systems in 56 stable refugee camps indicate wide variation in condom distribution and provision of post-exposure prophylaxis for HIV as part of clinical management of rape—both key activities of the MISP (unpublished observations, Whitmill, Tomczyk, Blanton, Doraiswamy, Haskew, Cornier, Schilperood, Spiegel). Regarding evaluation of reproductive health programming in humanitarian settings, Casey’s systematic review found no peer-reviewed papers that evaluated MISP implementation comprehensively since 2004 [13].

### Maternal and newborn health

Progress in maternal and newborn health followed a similar trajectory. In 2004, the global evaluation found that some maternal and newborn health services were available in stable refugee settings, but implementation of antenatal care and emergency obstetric care lagged [7]. The 2014 review documented important progress in advancing maternal and newborn health and its integration into primary health care services. Maternal and newborn care comprised the largest proportion (56%) of all reproductive health components in humanitarian health appeals from 2009 to 2013 [16]. It was also the most funded, receiving 56% of requested funds, and received the most amount of absolute funds—$684.8 million USD. Casey’s systematic review identified seven papers that described evaluations of humanitarian maternal and newborn health programs since 2004, demonstrating that some evaluation is occurring [13]. Preliminary findings from the study of 56 UNHCR refugee camps across ten countries show that screening for syphilis as part of antenatal care—a significant gap in the 2004 evaluation—increased over time in two countries and was consistently high in a third, although most camps did not meet desired screening levels across settings (unpublished observations, Whitmill, Tomczyk, Blanton, Doraiswamy, Haskew, Cornier, Schilperood, Spiegel).

Despite this progress, gaps remain, particularly regarding emergency obstetric and newborn care. A closer analysis of the trends in humanitarian appeals divulged that antenatal and postnatal activities were more frequently mentioned in proposals than emergency obstetric care [16]. Although Krause et al. found emergency obstetric and newborn care largely in place in the two settings assessed in Jordan [14], Casey et al. determined that only one of five hospitals and one of 58 health centers across three humanitarian settings met the criteria to adequately provide comprehensive and basic emergency obstetric and newborn care, respectively [15].^d^ These differences across settings likely reflect the relative availability of these services prior to the crisis. Indeed, the availability of this care in Jordan is unsurprising given its advanced health care system as opposed to the weak health systems in Burkina Faso, DRC, and South Sudan. Preliminary findings from the study of 56 refugee camps in ten countries show that none of the countries met the standard for proportion of live births performed by caesarian section (5% to 15%), and all but two countries were far below the standard of 100% of all births attended by a skilled health worker (unpublished observations, Whitmill, Tomczyk, Blanton, Doraiswamy, Haskew, Cornier, Schilperood, Spiegel). Further, maternal and infant deaths appeared to be underreported across all ten countries.

### Comprehensive abortion care

Comprehensive abortion care, which includes post-abortion care and safe abortion, is an essential component of reproductive health and is particularly important in settings with limited access to family planning and vulnerability to sexual violence—both of which characterize many humanitarian contexts. Safe abortion saves lives: UNFPA estimates that 25% to 50% of maternal deaths in refugee settings are due to complications of unsafe abortion [22]. Yet abortion-related services have been historically neglected in humanitarian responses, in part due to their highly politicized nature as well as health providers’ and communities’ misconceptions of the restrictiveness of national law [23, 24]. Indeed, the 2004 evaluation found limited post-abortion care available and safe abortion was not assessed [7]. Ten years later, the 2014 review documented some improvements in post-abortion care but a critical dearth of access to safe abortion within the extent of national law remained.

In Jordan, post-abortion care was generally available for Syrian refugees in the two settings assessed [14]. Casey et al.’s three-country study discovered that all assessed hospitals met the criteria to adequately provide post-abortion care, although availability was variable among health centers [15]. Still, post-abortion care was severely lacking in humanitarian health appeals from 2002 to 2013 [16], and the systematic review found no published studies that evaluated any component of comprehensive abortion care [13]. Further, safe abortion was neglected across all studies in terms of funding, evaluation, and implementation [13–16], although Krause et al. did find abortion available, within Jordan’s legal framework, in two hospitals [14].

### Family planning

Comprehensive family planning services, which can avert up to 32% of maternal deaths and almost 10% of childhood deaths [12], have also long been overshadowed by other pressing health needs. In 2004, the IAWG evaluation found relatively wide availability of short-acting methods, specifically oral contraceptive pills and injectables [7]. However, long-acting and permanent methods were lacking, despite evidence that a wide choice of methods raises overall contraceptive use and can enhance cost-effectiveness of programming [25, 26]. Emergency contraception as a method of family planning was not assessed in the 2004 evaluation.

Despite more concerted efforts to bring family planning to the fore, such as IAWG’s 2010 statement highlighting family planning as a life-saving intervention in humanitarian settings [27], most of the studies from the current review reflected limited progress relative to the other components of reproductive health since 2004. Tanabe et al. found that, of the reproductive health components in humanitarian health appeals from 2009 to 2013, only 14.9% contained family planning, the smallest of all components assessed; long-acting and permanent methods were rarely mentioned [16]. Of the 63 facilities assessed in Burkina Faso, DRC, and South Sudan, many provided pills and injectables, but again, emergency contraception as a method of family planning and long-acting and permanent methods were scarce [15]. Six of the 36 papers identified in Casey’s systematic review evaluated family planning programming, although all but one focused on short-acting methods [13]. At the same time, the MISP study in Jordan did find wide availability of IUDs in addition to short- acting methods [14], suggesting these methods are more likely to be provided in settings where they are already commonly used.

### Gender-based violence

In 2004, gender-based violence was an emerging area and, unsurprisingly, the weakest of the reproductive health components assessed in the global evaluation [7]. Gender-based violence is a broad field; reproductive health actors are responsible for providing good quality clinical care for survivors of sexual violence and ensuring protection measures are in place so clients can safely access services. The field has matured and expanded significantly over the past decade. Indeed, the 2014 review documented considerable progress in terms of funding, policies, and programming, yet program evaluation, prevention efforts, and systematic, comprehensive clinical management for rape survivors remained limited [13–15].

Preliminary findings from the UNHCR Health Information System study show that, although rape appeared to be underreported, five of the ten countries consistently met the standard of 100% of eligible survivors receiving post-exposure prophylaxis within 72 hours of an assault to minimize HIV transmission (unpublished observations, Whitmill, Tomczyk, Blanton, Doraiswamy, Haskew, Cornier, Schilperood, Spiegel). Gender-based violence, as related to reproductive health programming, comprised the second highest (46%) of all reproductive health proposals in humanitarian health and protection appeals from 2009 to 2013, a total increase of 34% over five years [16]. This suggests increasing attention to gender-based violence by implementing agencies. Donor support to gender-based violence totaled $308.9 million USD, a significant amount, although only 37% of the total request was met over the five-year period [16].

The other studies highlighted concerning gaps. The MISP assessment in Jordan found weak protection measures against sexual violence generally and only one assessed site had skilled staff and sufficient supplies to provide clinical care for rape survivors [14]. In the evaluation of reproductive health services across three settings, only three out of 63 total facilities met the criteria to adequately provide *selected* elements^e^ of clinical management of rape, and it was unclear whether these three facilities provided all components of the minimum package of post-rape treatment [15]. The systematic review found a plethora of descriptive papers that reported prevalence and types of sexual violence since 2004—which were noticeably lacking a decade ago—but none that evaluated the effectiveness of clinical management of rape services [13]; a 2013 evidence review of health in humanitarian settings similarly found extremely limited research on gender-based violence programming [28].

### HIV and other STIs

While the area of gender-based violence is expanding, HIV in emergencies has long been a field in its own right. HIV has historically received disproportionate more funding and attention relative to other reproductive health areas [29]. Indeed, preliminary findings from the long-term funding trend analysis show that the upsurge in total official development assistance-related reproductive health disbursements was largely due to a substantial increase in HIV funding (unpublished observations, Patel, Dahab, Tanabe, Murphy, Ettema, Guy, Roberts). Examining humanitarian appeals, Tanabe et al., however, reported a leveling of the field: since 2009, proposals that include HIV have declined whereas funding for other reproductive health areas has increased [16].

The 2004 global evaluation found uneven availability of condoms and STI treatment as well as very limited coverage of anti-retroviral therapy for people living with HIV [7]. Now, ten years later, the 2014 review identified some progress in the settings assessed, particularly regarding prevention of mother-to-child transmission and anti-retroviral therapy, but gaps generally mirrored those from a decade ago. Krause et al. and Casey et al. found sporadic availability of HIV and other STI services across four settings [14, 15]; anti-retroviral therapy was available at large referral hospitals, but rarely at primary care level, despite its inclusion as an addition to the MISP in the 2010 *Field Manual* and as a minimum standard in the *IASC Guidelines for Addressing HIV in Humanitarian Settings*[12, 30]. Preliminary findings from UNHCR’s Health Information System data also showed inconsistent provision of condoms in 56 refugee camps from 2007 to 2013 (unpublished observations, Whitmill, Tomczyk, Blanton, Doraiswamy, Haskew, Cornier, Schilperood, Spiegel). Casey identified an abundance of studies that evaluated anti-retroviral programs, which generally found that patient outcomes in conflict and post-conflict settings are comparable to stable settings [13], suggesting that we know what works but need to do it more systematically. Attention to HIV continued to overshadow other STI services across all studies, even though untreated STIs can lead to complications in pregnancy, infertility, reproductive cancers, and enhanced transmission of HIV.

### Additional findings

Preliminary findings from a survey of development and humanitarian organizations mirrored the findings above. The humanitarian and development agencies surveyed reported increased attention to MISP and all technical areas since 2004 as well as more efforts to address disaster risk reduction, accountability, and inter-agency coordination. They also reported, however, limited attention to safe abortion and cervical cancer screening and treatment (unpublished observations, Tran, Dawson, Meyers, Krause, Hickling).

Two notable findings from the 2004 evaluation were the lack of reproductive health services for adolescents and internally displaced, as opposed to refugee, populations [7]. In the 2014 review, some progress was evident with Krause et al. finding reproductive health services, with the exception of family planning for unmarried adolescents, relatively accessible for adolescent Syrian refugees in Jordan [14]; Casey identified five papers that evaluated adolescent HIV programs in humanitarian settings [13]. Yet few adolescent-friendly services were in place across the three settings assessed by Casey et al. [15], and Tanabe et al. found limited mainstreaming of adolescents in health and protection-related funding proposals [6]. Grey literature also documents limited availability of adolescent reproductive health programs in humanitarian settings [31].

Regarding reproductive health services for internally displaced persons, the 2004 evaluation and other research primarily focused on maternal health, found better reproductive health outcomes in stable refugee camp settings than in neighboring host communities or in refugees’ home countries [7, 32–34]. In the selected settings for the 2014 review, the studies established that, although services for Syrian refugees in Jordan were markedly more available and accessible than for those internally displaced in DRC, services for refugees in camps in South Sudan were comparatively less available and of poorer quality. Among Malian refugees in Burkina Faso, some reproductive health services, such as STI care and prevention-of-mother-to-child transmission of HIV, were more consistently available at government-run clinics for the surrounding host population than in camp health centers. In the assessed settings, availability and access were contingent on a complex constellation of factors, such as humanitarian space, funding dedicated to the reproductive health response, the robustness of the setting’s health system, and whether services were commonly available before the emergency.

One of the more striking and urgent findings of the 2014 evaluation was that, even when reproductive health services were in place, uptake of many services lagged across all settings. Many affected communities were unaware of existing services or did not know of their benefits. Even those who could identify advantages—such as accessing post-exposure prophylaxis for HIV within 72 hours after rape—reported that communities largely shunned services, citing socio-cultural barriers such as shame and anxieties about possible social sanctions. This finding challenges the “if you build it, they will come” assumption that at times permeates health programming. Reproductive health refracts cultural sensitivities, and effective programming requires community mobilization activities, particularly for services to which the community may not have previously had access. IAWG has made some inroads in recognition of this, such as the inclusion of “ensuring community awareness of available services” as an activity of the MISP [12] and the development of behavior change communication materials [35]. Notably, Casey et al. documented a significant uptake of facility-based delivery services across three settings as a result of outreach by health actors, highlighting that behavior change to increase use of reproductive health services is possible with the appropriate strategy.

Many of the other challenges identified in these studies are long-standing. Poor commodity management and security were key barriers to good quality care. Negative provider attitudes and behaviors, such as disrespect towards women seeking family planning services, hindered some people from seeking care. Restrictive policies and misinformation about existing policies prevented implementation of critical services, and poor quality data collection undermined service monitoring. Some gaps in care resulted from a dearth of skilled staff. Although integration of reproductive health into comprehensive primary health care was evident in some sites, further efforts are needed for effective integration across all levels of care.

## Considerations for future directions

The studies for the 2014 IAWG global review documented considerable progress in the field since the previous evaluation a decade ago. Funding and awareness have increased significantly, and service provision has expanded. However, programmatic needs continue to outweigh financial support, implementation is not systematic and is of variable quality, and evidence for program efficacy remains scarce. The review spotlighted poor commodity management and security, limited availability of comprehensive abortion care, and lack of community mobilization to increase reproductive health service uptake as particularly critical gaps.

The Nobel Prize-winning economist Elinor Ostrom imagined the ideal aid system as one that would “reward people for developing imaginative ideas that draw on the complexity of the real world, that leave people in developing countries more autonomous, less dependent, and more capable of crafting their own future” [36]. She proposed that, in a rapidly evolving global society, the nature of change is non-linear and is achieved not through pre-fabricated solutions but through creating adaptive, dynamic systems. Many of the strategies used by IAWG and other actors advancing reproductive health on the humanitarian agenda align with Ostrom’s complexity approach. Their efforts have often been innovative, responsive, and multi-faceted, involving local communities up to the highest levels of international bodies and engaging with all phases of the emergency management cycle.

The way forward would benefit from applying dynamic approaches that knit together disparate elements of the emergency management universe, including pre-crisis preparedness and risk reduction efforts, crisis response interventions, and early recovery and rehabilitation activities. Humanitarian response must be integrated as an essential piece of health systems work and reproductive health as an essential component of health. Indeed, the findings from the 2014 review indicate that the relative robustness of the pre-existing health care system and the availability of reproductive health services before an emergency determine the availability and uptake of these services during the response and recovery. Long before a crisis, donors, UN agencies, governments, and international and national NGOS, among others, must support the capacity of Ministries of Health and Disaster Management, national and community-based organizations, health workers, and communities themselves to strengthen health systems with an emphasis on comprehensive reproductive health, accessibility, and resilience-building. This includes, for example, funders supporting community involvement in the design and delivery of reproductive health services, national governments addressing policy barriers to reproductive health service implementation, and national medical and nursing schools integrating reproductive health into their curricula. Humanitarian and development agencies addressing reproductive health must engage, coordinate, and reinforce each others’ work, and donors are called upon to support cohesive programming that integrates both pre- and post-crisis efforts. With the increasing urbanization of displacement—as demonstrated in Krause et al.’s study—implementing agencies need to adapt and develop appropriate operational frameworks and forge new relationships with municipal authorities and urban service providers. Preliminary findings from the institutional capacity survey indicate that an increasing number of stakeholders are addressing emergency preparedness, disaster risk reduction, and recovery measures related to reproductive health, but much more effort is needed to systematically and sustainably bridge the humanitarian-development divide.

Casey et al. and Krause et al.’s studies highlight the urgent need to address reproductive health commodity security. Ministries of Health and health NGOs must strengthen commodity management processes to prevent stock-outs and provide consistent access to care. At the global level, with donor support, IAWG could link with the Reproductive Health Supplies Coalition and other development actors to spearhead a concerted effort to promote effective reproductive health supply chain management from the onset of a crisis response throughout recovery.

The significant growth in funding for and implementation of gender-based violence and HIV programming, as found by Tanabe et al., illustrates the expansion of these fields over the past decade. As such, the protection and health clusters/sectors, led by UNHCR and WHO, respectively, as well as the gender-based violence area of responsibility, co-led by UNFPA and UNICEF, must strengthen coordination and delineation of roles to support an aligned approach and integrated interventions. Gender-based violence and HIV focal points ought to maintain linkages with reproductive health actors to ensure a harmonized response by, for example, attending respective coordination meetings in the field. Indeed, the lynchpin for an effective reproductive health humanitarian response is sustained, inter-agency reproductive health coordination with a designated lead agency; funders are obliged to support this type of coordination and not just direct service provision.

Casey et al. and Krause et al.’s studies demonstrate the need to enhance providers’ knowledge base and address personal beliefs that affect professional conduct. Service providers should explore competency-based clinical trainings on reproductive health within a rights-based framework. These two studies also show the importance of a coherent transition from MISP to comprehensive reproductive health services, as outlined in the *Granada Consensus*[11]. Preliminary findings from the Health Information System study as well as Casey et al.’s study indicate that UNHCR and implementing agencies must strengthen their data collection and management. To enhance the weak evidence base as identified in the literature review, academic institutions could spearhead more systematic research and program evaluation to identify better ways to serve the reproductive health needs of crisis-affected communities.

In order to realize Ostrom’s vision for aid, UN agencies, donors, and international NGOs must listen to, engage, and work with local, national, and government agencies in a way that addresses power dynamics and promotes ownership and leadership. The 2014 review clearly highlights that humanitarian and development actors must identify and develop effective strategies to meaningfully engage affected communities to increase use of reproductive health services, meet their reproductive health needs, and augment participation in the programs that affect their lives. Implementing agencies can explore contextually appropriate new technologies, such as social media and mobile technologies, to increase two-way communication with communities [37].

The studies also bring to the fore the marked lack of attention to adolescent reproductive health in terms of funding, access to services, programming, and program evaluation; donors and implementing agencies must prioritize adolescents as well as other marginalized groups, such as people with disabilities, sex workers, elderly, and lesbian, gay, bisexual, and transgender persons, to ensure they access and enjoy good quality reproductive health care. In addition, Casey et al.’s research in Burkina Faso, DRC, and South Sudan sheds light on the challenges of providing good quality care in remote settings with limited health providers; more attention to task-sharing to address human resource gaps and to developing alternative service delivery models can help facilitate the provision of services even in the most hard to reach areas. These and other efforts should be underpinned by humanitarian principles and grounded in a culture that fosters accountability, learning, adaptation, and flexibility.

Tanabe et al.’s study as well as preliminary findings from the long-term trend analysis of official development assistance demonstrate the need for increased funding for reproductive health in crises. Donor governments such as Australia, Belgium, and the U.S. have thankfully stepped up in recent years to support humanitarian reproductive health and protection programming, but more donor champions are required to close the funding gap, ensure equitable funding across reproductive health areas, and support fluid, innovative humanitarian programs rather than short-term, quantifiable interventions.

Multi-sectoral efforts to advance reproductive health in crises are also needed at the global level. The architects of the Sustainable Development Goals, for example, should integrate comprehensive reproductive health care for communities affected by humanitarian crises into the post-2015 agenda. Reproductive health and gender issues remain on the periphery of climate change adaptation and mitigation planning, and climate change leaders should ensure reproductive health actors are at the table.

There is no panacea to addressing reproductive health needs in increasingly complex humanitarian crises. But we do know that effective humanitarian action is contingent on the capacity, ability, and desire of agencies to work together [36]. Collaborative efforts that embrace holistic, adaptive approaches, such as IAWG at the global level and many national and community-based networks at the field level, are critical to continue to effectively move the agenda forward. These partnerships need to be supported and consolidated. Indeed, sustained, dedicated, predictable funding to maintain the coordination of IAWG is essential as it leads the coordinated effort to protect and promote the sexual and reproductive well-being of communities affected by humanitarian crises around the world.

## Authors' information

The author has been a member of the Inter-agency Working Group on Reproductive Health in Crises since 2003.

## Endnotes

^a^ Formerly known as the Inter-agency Working Group for Reproductive Health in Refugee Situations^b^ The MISP is a set of priority activities designed to ensure coordination, prevent sexual violence and provide care for survivors, prevent maternal and neonatal morbidity and mortality, and HIV transmission, and plan for comprehensive reproductive health services. Additional priorities include ensuring the availability of contraceptives to meet demand, syndromic treatment of STIs, anti-retrovirals for continuing users, and menstrual hygiene supplies.^c^ Formerly known as the Reproductive Health for Refugees Consortium, the RHRC Consortium is comprised of the American Refugee Committee, CARE, Columbia University, International Rescue Committee, JSI Research and Training Institute, Marie Stopes International, and the Women's Refugee Commission.^d^ Health centers should provide basic emergency obstetric and newborn care, which includes: 1. administering parenteral antibiotics; 2. administering uterotonic drugs; 3. administering parenteral anticonvulsants (e.g., magnesium sulphate); 4. performing manual removal of placenta; 5. performing removal of retained products of conception (e.g., manual vacuum aspiration); 6. performing assisted vaginal delivery (e.g., vacuum extraction); 7. performing neonatal resuscitation (with bag and mask). Hospitals should provide comprehensive emergency obstetric and newborn care, which includes the seven functions outlined above as well as: 8. performing blood transfusion; and 9. performing surgery (e.g., Caesarean section).^e^ The selected elements of clinical management of rape assessed were the availability of emergency contraception, post-exposure prophylaxis for HIV, and antibiotics for prevention of sexually transmitted infections; the provision of these drugs in the previous three months; and at least one staff trained to provide clinical management of rape. The full minimum package of clinical management of rape for low-resource settings includes 25 elements [38].

## List of abbreviations

DRC: Democratic Republic of the Congo
IAWG: Inter-agency Working Group on Reproductive Health in Crises
ICPD: International Conference on Population and Development
MISP: Minimum Initial Service Package (for Reproductive Health)
NGO: Nongovernmental organization
RAISE: Reproductive health Access, Information and Services in Emergencies
RHRC Consortium: Reproductive Health Response in Conflict Consortium
SPRINT: Sexual and Reproductive Health Programme in Crisis and Post-Crisis Situations
STI: Sexually transmitted infection
UNFPA: United Nations Population Fund
UNHCR: United Nations High Commissioner for Refugees
WHO: World Health Organization.
